# Supplementary material for: Regulatory Mechanisms of Exogenous Acyl-Homoserine Lactones in the Aerobic Ammonia Oxidation Process Under Stress Conditions
Source: Microorganisms. 2025 Mar 14;13(3):663. doi: 10.3390/microorganisms13030663 (PMC11946751; doi:10.3390/microorganisms13030663)
Supplement: Supplementary file 1 [file microorganisms-13-00663-s001.zip › microorganisms-3497738-supplementary.pdf]

Supplementary Materials

Regulatory Mechanisms of Exogenous Acyl-Homoserine Lactones in the Aerobic Ammonia Oxidation Process Under Stress Conditions

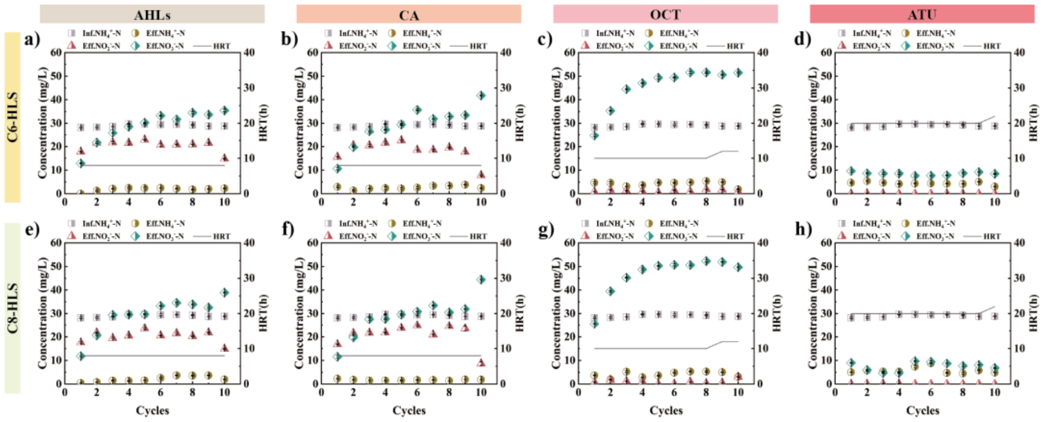

Figure S1. Nitrogen changes in incoming and outgoing water during each cycle of the experiment.

Table S1. Composition of synthetic wastewater

Composition	Concentration
NH_4Cl	214 mg/L
$NaHCO_3$	672 mg/L
KH_2PO_4	140 mg/L
$MgSO_4 \cdot H_2O$	300 mg/L
$CaCl_2$	140 mg/L
Trace element	1 mL/L

Table S2. Composition of trace elements in synthetic wastewater

Composition	Concentration
$Na_2EDTA \cdot 2H_2O$	4780 mg/L
$FeCl_2 \cdot 4H_2O$	1988 mg/L
$MnCl_2 \cdot 4H_2O$	104 mg/L
$NiCl_2 \cdot 6H_2O$	24 mg/L
$CoCl_2 \cdot 6H_2O$	24 mg/L
$CuCl_2 \cdot 2H_2O$	17 mg/L
$ZnCl_2$	68 mg/L
$Na_2MoO_4 \cdot 2H_2O$	24 mg/L
$Na_2WoO_4 \cdot 2H_2O$	33 mg/L
H_3BO_3	62 mg/L
